# Development and Psychometric Evaluation of the Nursing Care Motivation Scale: A Methodological Study

**DOI:** 10.1155/jonm/7716129

**Published:** 2026-08-31

**Authors:** Yeter Kurt, Ebru Turhal, Havva Öztürk, Aleyna Bülbül

**Affiliations:** ^1^ Fundamentals of Nursing Department, Faculty of Health Sciences, Karadeniz Technical University, Trabzon, Türkiye, ktu.edu.tr; ^2^ Medical Education and Training Simulation Center, Faculty of Medicine, Karadeniz Technical University, Trabzon, Türkiye, ktu.edu.tr; ^3^ Management of Nursing Department, Faculty of Health Sciences, Karadeniz Technical University, Trabzon, Türkiye, ktu.edu.tr; ^4^ Internal Medicine Nursing Department, Institute of Health Sciences, Karadeniz Technical University, Trabzon, Türkiye, ktu.edu.tr

**Keywords:** motivation, nurses, nursing care, patient care

## Abstract

**Background:**

Nursing care motivation is a key determinant of caring behaviours and quality of care outcomes. However, existing instruments do not adequately capture motivation specific to the nursing care context.

**Objective:**

To develop a valid and reliable instrument to determine nurses’ motivation and willingness to provide care, as well as the factors guiding their caring behaviour.

**Design:**

Instrument development and psychometric testing of content validity, reliability and construct validity.

**Methods:**

This methodological study included 30 nurses for the pilot study, 397 for principal component analysis (PCA), 350 for confirmatory factor analysis (CFA) and 30 for test–retest reliability. Face and content validity, construct validity and reliability analyses were conducted to develop the scale and assess its psychometric properties. Data were collected between December 2024 and December 2025.

**Results:**

The content validity index of the scale was 0.98. PCA revealed a five‐factor structure with 27 items, explaining 72.35% of the total variance. Factor loadings ranged from 0.65 to 0.89. CFA supported the model with acceptable fit indices (*χ* ^2^/df = 2.446, CFI = 0.94, TLI = 0.93 and RMSEA = 0.06). The Cronbach’s *α* coefficient of the scale was 0.952, the Guttman split‐half was 0.843, and the test–retest reliability was 0.937.

**Conclusion:**

The Nursing Care Motivation Scale (NCMS) is a valid and reliable tool that can be used by nurses.

**Implication for Nursing Management:**

The NCMS provides nurse managers with a practical tool to identify modifiable factors influencing nursing care motivation, inform targeted interventions and enhance the quality and sustainability of patient‐centred care.

## 1. Introduction

Caring is considered the expression of nursing and caring constitutes the essence of nursing practice [1]. It is not merely a technical activity; rather, it represents a concrete manifestation of the meaning that nursing attributes to humanity, professionalism and the act of providing care. Caring is shaped through various individual and internal processes experienced by nurses [2]. The World Health Organization (WHO) and national health authorities also emphasize that, for the sustainable delivery of high‐quality care, professional competencies should include not only technical knowledge and skills but also psychosocial and motivational factors that nurture the willingness to provide care [3]. This emphasis demonstrates that the nurse’s motivation holds a central position among the factors determining the quality of care. From a theoretical perspective, nursing care motivation cannot be adequately explained by general work motivation alone. Contemporary nursing and motivational theories conceptualize care motivation as a multidimensional construct encompassing professional values, caring relationships, ethical responsibility, autonomy, competence, relatedness and the meaning attributed to providing care. In particular, Watson’s Human Caring Theory [4] emphasizes the humanistic and relational dimensions of caring, Benner’s Novice to Expert Model [5] highlights the role of professional identity and clinical competence in shaping nurses’ motivation and Self‐Determination Theory (SDT) [6] explains how the fulfillment of autonomy, competence and relatedness promotes intrinsic motivation for caring. Together, these theoretical perspectives provide a comprehensive framework for understanding the complex nature of nursing care motivation.

A key source of motivation that enables nurses to remain in their profession despite the challenges they encounter is the sense of “meaning” derived from caring. This sense of meaning is nourished by several interrelated components, including spirituality as a universal sense of connectedness, a sense of pride and joy derived from helping others, supportive interpersonal relationships, a moral responsibility to contribute to the well‐being of others, and thriving, professional growth/success [7]. In this context, nursing care motivation represents a distinct motivational domain that differs from general occupational motivation by focusing specifically on the act of providing care. It arises from the desire to help others and to provide compassionate and beneficial care, serving as an internal driving force that brings energy, meaning, and continuity to nursing practice [8]. This conceptualization aligns with the notion of “care motivation” described by Moody and Pesut [9] as an inner drive grounded in values that initiates and directs human behaviour. Within this framework, nursing care motivation enables nurses to perceive caring not merely as a task that must be fulfilled but as a meaningful and humane process integrated with their professional identity. Accordingly, nursing care motivation extends beyond general occupational motivation by integrating professional, relational, ethical, organisational and psychological dimensions that are unique to nursing practice. Furthermore, nursing care motivation emerges as a key factor that directly influences the quality and continuity of nursing care behaviours. Positive experiences and professional satisfaction derived from the caring process enhance care motivation; in turn, higher levels of nursing care motivation are associated with more attentive, compassionate and empathetic care practices [3]. Similarly, Asadi et al. [8] reported that nurses with higher levels of nursing care motivation tend to establish more empathetic and supportive relationships with patients, respond more sensitively to patients’ needs and engage more actively in the care process. Moreover, within the dynamic and reciprocal nature of the caring relationship, the act of providing care extends beyond a mere professional obligation and evolves into an experience that fosters joy, meaning, self‐esteem and stronger interpersonal connections. This transformation contributes to positive psychosocial and professional outcomes for both care providers and care recipients [10]. It supports patients’ recovery and improves care quality.

Nurses’ motivation toward the caring process is widely recognized as a key determinant of both professional satisfaction and the quality of care delivered [11]. Given this multidimensional theoretical conceptualization, instruments designed to measure nursing care motivation should adequately represent these diverse domains. However, existing measurement tools have primarily been developed to assess general work motivation rather than motivation specifically related to providing nursing care. In the literature, nurses’ motivation has mostly been examined using instruments designed to measure general or work‐related motivation. Commonly used tools include the Situational Motivation Scale, the Work Motivation Measurement Scale for Nurses, the Multidimensional Work Motivation Scale and the Academic Motivation Scale [12, 13]. These instruments assess motivation through broad psychosocial and occupational dimensions such as intrinsic and extrinsic orientations, expectations related to reward and punishment, perceived job value, autonomy, perceived competence and organisational influences. Furthermore, these instruments are predominantly grounded in organisational or occupational motivation theories and therefore do not operationalize core concepts emphasized in nursing theories, such as caring relationships, professional identity, ethical commitment and the meaningfulness of providing care. However, they do not specifically capture the motivational processes directly associated with patient care, which constitutes the core of nursing practice. Indeed, numerous studies have evaluated nurses’ willingness to provide care or their motivation toward caring using self‐report questionnaires or general motivation scales. For example, the Nursing Motivation Scale, although developed to measure intrinsic and extrinsic motivation among nurses, does not explicitly address the specific motivational processes and clinical care dynamics that guide nurses in delivering patient care [14]. This suggests that existing job motivation scales are limited in their capacity to assess nursing care motivation as a multidimensional and context‐specific construct within clinical practice. Moreover, most of these instruments primarily focus on outcomes such as job satisfaction, organisational commitment and overall work motivation, and therefore do not comprehensively reflect key aspects of nursing care, including professional responsibility, therapeutic nurse–patient relationships, ethical sensitivity and clinical care processes. In contrast, the Nursing Motives for Helping Scale (N‐MHS) offers a more focused perspective on nursing care motivation by evaluating three motivational dimensions, altruistic motivation, reward‐seeking and punishment avoidance, based on Batson’s theory of helping motivation (nine items across three subdimensions) [15]. Although this scale provides valuable insight into nurses’ motivational orientations toward helping behaviour, it conceptualises nursing care motivation primarily within the framework of the desire to help. Consequently, it reflects only a limited aspect of the complex interplay between nursing care and factors such as professional identity, clinical responsibilities, patient outcomes, organisational conditions and emotional or spiritual dimensions of caring. This limitation has prompted researchers to develop alternative instruments tailored to specific clinical or research contexts in an effort to better capture the complexity of nursing care motivation.

In addition, several researchers have used self‐developed questionnaires to assess nurses’ motivation to provide care [8, 16]. Although these instruments have provided valuable evidence within specific research settings, their limited use across diverse populations and the lack of comprehensive psychometric validation have restricted their adoption as standardised measures of nursing care motivation. This limitation restricts the comparability of nursing care motivation across different clinical settings and makes it difficult to establish clear associations between nursing care motivation and the quality of care. An evaluation of the existing literature indicates that there are relatively few valid and reliable instruments capable of assessing nurses’ willingness to provide care, their intrinsic orientation toward the caring process, and the factors that sustain care‐related behaviours within a multidimensional clinical framework. Taken together, the existing literature reveals both conceptual and methodological gaps in the assessment of nursing care motivation. Conceptually, currently available instruments do not adequately reflect the multidimensional theoretical foundations proposed by contemporary nursing and motivational theories. Methodologically, few instruments have undergone comprehensive psychometric evaluation specifically for measuring nursing care motivation within clinical settings. Consequently, there remains a need for a theoretically grounded and psychometrically robust instrument capable of comprehensively assessing the professional, organisational, humanistic and psychological dimensions of nursing care motivation. Accordingly, the present study aimed to develop and psychometrically evaluate the Nursing Care Motivation Scale (NCMS), which was theoretically grounded in Watson’s Human Caring Theory, Benner’s Novice to Expert Model and SDT. The NCMS was designed to comprehensively assess the multidimensional motivational processes underlying nurses’ willingness to provide care. A valid and reliable scale for measuring nursing care motivation may assist clinicians, nursing administrators and researchers in assessing nurses’ willingness and motivation to deliver care, identifying factors that influence care motivation and developing targeted interventions and educational strategies to strengthen caring behaviours.

## 2. The Study

### 2.1. Aim

This study aimed to develop a scale to determine nurses’ motivation and willingness to provide care, identify the motivational dimensions and factors influencing their caring behaviour, and evaluate its psychometric properties.

## 3. Methods

### 3.1. Study Design

This methodological study aimed to develop and psychometrically evaluate the Nurses’ Care Motivation Scale (NCMS). This study was conducted in three sequential phases: (1) instrument development (construct definition and item generation); (2) content validity assessment (face validity, expert review and pilot testing) and (3) evaluation of the measurement properties (structural validity, construct validity and reliability). The study was conducted and reported in accordance with the COnsensus‐based Standards for the selection of health Measurement INstruments (COSMIN) recommendations to ensure methodological rigour and transparent reporting of the instrument development and psychometric evaluation process [17, 18]. Specifically, the COSMIN Risk of Bias Checklist for Instrument Development was followed step‐by‐step to evaluate content validity, structural validity and internal consistency, ensuring that the NCMS fulfills all quality criteria required to be selected as a robust outcome measurement instrument in healthcare research. In addition, the study was conducted and reported in accordance with the Strengthening the Reporting of Observational Studies in Epidemiology (STROBE) Statement [19]. Throughout the study process, the scale development guidelines proposed by Boateng et al. [20] and Morgado et al. [21] were also followed.

### 3.2. Instrument Development

#### 3.2.1. Construct Definition

The construct underlying this study was defined prior to item generation in accordance with COSMIN recommendations for instrument development. The construct of “nurses’ motivation to provide care” was conceptualized as a multidimensional psychological process that reflects nurses’ intrinsic and extrinsic motivational forces influencing their willingness, commitment and sustained engagement in providing high‐quality nursing care.

Within this conceptualization, care motivation was considered not as a unidimensional trait but as a dynamic construct shaped by professional identity, perceived competence, workplace conditions, perceived patient outcomes and spiritual fulfillment derived from caregiving. The theoretical foundation of the construct was established through an integrative synthesis of three major frameworks in nursing and motivational science: Watson’s Human Caring Theory, Benner’s Novice to Expert Model and SDT. First, Watson’s Human Caring Theory served as the primary theoretical foundation, particularly for developing items within the subdimensions of “Positive Care Outcomes (PCO)” and “Spiritual Satisfaction,” due to its holistic perspective emphasizing the humanistic, ethical, therapeutic and spiritual aspects of care [4]. Second, Benner’s Novice to Expert Model, which describes nurses’ progression in clinical decision‐making, autonomy, professional identity and competence development, provided the conceptual basis for the subdimensions Professional Identity and Values (PIV) and Sense of Achievement and Competence (SAC) [5]. Third, SDT, developed by Deci and Ryan, which explains both intrinsic and extrinsic motivational processes, was used to guide the conceptualization of several subdimensions of the scale. In particular, the theory’s three fundamental psychological needs—autonomy, competence and relatedness—were considered in the development of items within the subdimensions of “Supportive Care Conditions (SCC),” “Sense of Achievement and Competence,” “Positive Care Outcomes,” and “Spiritual Satisfaction” [6]. Based on these three theoretical approaches, the preliminary version of the NCMS was developed. The initial scale consisted of 42 items designed to evaluate five dimensions: SCC, PIV, PCO for the patient, SAC and Sense of Spiritual Satisfaction (SSS). These domains formed the conceptual basis for subsequent item generation.

To enhance theoretical transparency, a mapping process was conducted to align each NCMS subdimension with its corresponding theoretical foundations. Watson’s Human Caring Theory, Benner’s Novice to Expert Model and SDT jointly informed the conceptual structure of the scale. Specifically, the basic psychological needs of SDT (autonomy, competence and relatedness) were systematically mapped onto the relevant subdimensions of the NCMS to ensure conceptual alignment between theory and item generation (Table 1).

**TABLE 1:** Mapping of theoretical frameworks to NCMS subdimensions and item development.

Theoretical framework	Key concept	Contribution to NCMS	Representative item focus
Watson’s Human Caring Theory	Humanistic caring, transpersonal relationships, meaning of care	Positive Care Outcomes; Spiritual Satisfaction	Compassionate care, finding meaning in caring, emotional fulfilment

Benner’s Novice to Expert Model	Professional development, clinical expertise	Professional Identity and Values; Sense of Achievement and Competence	Clinical expertise, professional growth, achievement

Self‐Determination Theory	Autonomy	Supportive Care Conditions	Decision‐making autonomy, independence in care
	Competence	Sense of Achievement and Competence; Positive Care Outcomes	Feeling capable, effective patient care
	Relatedness	Supportive Care Conditions; Spiritual Satisfaction	Therapeutic relationships, connectedness, belonging

#### 3.2.2. Item Generation

A systematic literature review was performed using ScienceDirect, PubMed, Web of Science, Google Scholar and ULAKBİM databases. The search strategy included the keywords “nursing care,” “motivation,” “caring motivation,” “professional motivation,” “nursing work environment,” “caring outcomes” and “caring behaviours.” In addition to the literature review, previously developed measurement instruments related to nursing motivation, caring behaviours, job motivation and professional engagement were examined to ensure comprehensive item coverage and conceptual saturation. Based on the synthesized findings, an initial item pool was generated by the research team. Items were developed to reflect both theoretical constructs and empirical findings from the literature.

To establish a rigorous conceptual foundation, the item pool was structured according to the dimensions of the O∗NET Content Model (e.g., worker characteristics, occupational requirements and work context)[22] and mapped onto five predefined conceptual domains derived from the theoretical framework: Supportive Care Conditions, PIV, PCO for the patient, SAC and SSS.

During item construction, attention was given to ensuring that each item reflected a single clear idea, used simple and unambiguous language, and was appropriate for the clinical context of nursing practice. Redundancy was minimised, and items were formulated to capture both intrinsic and extrinsic motivational aspects of care provision.

To integrate contemporary clinical reality into the item generation phase and secure face validity, a clinical expert focus group panel was convened. This panel consisted of five registered nurses with extensive clinical backgrounds, including three expert intensive care nurses and two experienced ward nurses. During this collaborative session, the panel actively reviewed the draft items against the O∗NET framework, refined the phrasing to align with active nursing terminology, and proposed new items addressing real‐world clinical workloads, organisational dynamics and concrete barriers to care motivation. As a result of this empirical refinement, a preliminary version of the Nurses’ Care Motivation Scale (NCMS) consisting of 42 items was developed. This initial version represented the theoretical, empirical and clinical integration of nursing motivation literature and formed the basis for subsequent content validity evaluation.

### 3.3. Content Validity

#### 3.3.1. Face Validity

Consistent with COSMIN recommendations, content validity was first assessed by evaluating the comprehensibility of the draft items through feedback from representatives of the target population and academic experts. To assess the face validity of the draft NCMS, feedback was obtained from both peers (five nurses) and experts (ten academic nurses). Based on their qualitative suggestions and quantitative consensus on item comprehensibility, 19 items in the draft scale were revised to improve spelling, punctuation and clarity without altering their intended meaning. In addition, the scale was reviewed by an expert in the Turkish language and literature to ensure linguistic accuracy and clarity.

#### 3.3.2. Expert Review

The relevance, comprehensiveness and comprehensibility of the draft scale were further evaluated through expert review in accordance with COSMIN recommendations for content validity.

The draft scale was sent via e‐mail to 10 academic nurses with expertise in the fields of nursing, fundamentals of nursing, nursing management and nursing education. The items were evaluated using the Davis [23] technique. Experts assessed each item in terms of clarity, relevance to the study objective, discriminative ability and cultural appropriateness. Each item was rated on a four‐point scale: “appropriate,” “item requires minor revision,” “item requires major revision,” and “item is not appropriate.” Experts independently completed the evaluation forms, and all ratings were collected individually to ensure independent judgement. Based on expert evaluations, the item‐level content validity index (I‐CVI) was calculated for each item using the proportion of experts rating the item as either “appropriate” or “item requires minor revision”. According to the criteria proposed by Polit and Beck [24], items with an I‐CVI value below 0.78 were considered to have inadequate content validity; therefore, one item was removed from the draft scale. The scale‐level CVI based on the average method (S‐CVI/Ave) for the remaining 41 items was 0.98, indicating excellent content validity.

Furthermore, to account for chance agreement among the experts, the probability of chance agreement (Pc) and the modified kappa coefficient (k^∗^) were calculated for each item. The modified kappa values for the retained items ranged from 0.79 to 1.00, indicating excellent agreement beyond chance.

#### 3.3.3. Cognitive Pretesting (Pilot Study)

Following content validation, cognitive pretesting was conducted to evaluate the clarity, comprehensibility, feasibility, and administration time of the draft scale before large‐scale psychometric testing.

A pilot study was conducted with 30 nurses to evaluate the clarity of the scale items and the average response time, as well as to perform preliminary psychometric analyses. These nurses were not included in the main study sample. The inclusion criteria for the pilot study were identical to those applied in the main study to ensure methodological consistency across study phases. As a result of the pilot testing, no major comprehension difficulties or ambiguous items were identified by participants. Minor wording refinements were made in several items to improve clarity without changing their conceptual meaning. However, no items were removed from the scale, as all items were considered conceptually relevant and had already demonstrated adequate content validity in the expert review phase. The Cronbach’s alpha coefficient for the 41‐item scale was calculated as 0.95.

### 3.4. Evaluation of Measurement Properties

Following the COSMIN taxonomy for psychometric evaluation, measurement properties were systematically assessed across specific sequential subsections comprising item analysis, structural validity, hypothesis testing (convergent/discriminant validity) and reliability.

#### 3.4.1. Preliminary Item Analysis

In accordance with COSMIN recommendations, before executing structural validity testing, a preliminary item analysis was performed on the first dataset (*n* = 397) to evaluate item performance. Item discrimination was examined using the critical ratio method, comparing the highest and lowest scoring 27% of participants. Items with *t*‐values < 3.0 or *p* ≥ 0.05 were targeted for removal. Item–total correlations were assessed using Pearson’s correlation coefficient (r); items with *r* < 0.30 were excluded to maintain internal consistency and ensure that all items contributed sufficiently to the target construct.

All 41 items successfully fulfilled the preliminary item analysis criteria (*t* > 3.0, *p* < 0.001; *r* ≥ 0.30), and thus, the exploratory factor analysis was initiated with 41 items, which was subsequently optimised to 27 items for the confirmatory factor analysis (CFA) phase.

#### 3.4.2. Construct Validity and Construct Validity Assessment

The construct and structural validity of the NCMS was evaluated through a sequential assessment of structural validity and hypothesis testing for convergent and discriminant validity. Prior to factor analyses, data screening procedures were conducted to ensure the appropriateness of the dataset for multivariate analysis. Skewness and kurtosis values were examined for each item, and all values were found to be within the acceptable range of ±2, indicating normal distribution. In addition, the Kaiser–Meyer–Olkin (KMO) measure of sampling adequacy, Bartlett’s test of sphericity, and the anti‐image correlation matrix were examined to determine the suitability of the data for factor analysis.

Exploratory factor structure was initially examined using principal component analysis (PCA) as an extraction method to identify the underlying dimensional structure of the scale [25]. Although common factor extraction methods are generally preferred in scale development [26], PCA was adopted at this preliminary stage because the primary aim was data reduction—identifying a parsimonious subdimensional structure from a large pool of draft items—prior to establishing the measurement model via CFA [25]. Given the high total explained variance (> 70%) and strong item communalities (> 0.50), PCA yields results empirically equivalent to common factor extraction methods under these optimal data conditions [27, 28]. Given the theoretical assumption that the dimensions of care motivation are interrelated, Promax oblique rotation was applied to allow for correlations among factors [29].

The number of factors was determined based on eigenvalues greater than 1 and inspection of the scree plot [30]. Items with factor loadings below 0.50 were removed from the scale, whereas items with loadings ≥ 0.50 were retained due to their adequate representation of the underlying construct [31]. In addition, the rotated pattern matrix was examined to evaluate cross‐loadings. Items loading ≥ 0.40 on more than one factor or showing conceptually ambiguous loading patterns were considered for removal to achieve a simple and interpretable factor structure. Item retention decisions were based on both statistical criteria and conceptual consistency.

Following exploratory analysis, CFA was conducted using AMOS 21.0 to validate the factor structure identified in the PCA. Although the NCMS items are measured on a 5‐point Likert scale and therefore represent ordinal data, continuous‐variable maximum likelihood (ML) estimation was applied, as univariate skewness and kurtosis values for all items were strictly within the acceptable range of ±2. Continuous‐variable ML estimation is psychometrically robust and yields accurate parameter estimates for ordinal data when distributional thresholds are satisfied and the scale features five or more response categories [32, 33]. Model fit was evaluated using multiple goodness‐of‐fit indices, and modification indices were considered only when theoretically justified. Model fit was evaluated using multiple goodness‐of‐fit indices, and modification indices were considered only when theoretically justified. In line with COSMIN guidelines, convergent and discriminant validity were evaluated as part of construct validity. Composite Reliability (CR) and Average Variance Extracted (AVE) were calculated according to the formulas proposed by Fornell and Larcker [34]. Convergent validity was considered adequate when CR was ≥ 0.70 and AVE was ≥ 0.50. Discriminant validity was evaluated using the Fornell–Larcker criterion and the Heterotrait–Monotrait Ratio of Correlations (HTMT). Adequate discriminant validity was established when AVE exceeded both the Maximum Shared Variance (MSV) and Average Shared Variance (ASV) and all HTMT values < 0.85 [34, 35].

#### 3.4.3. Reliability and Internal Consistency

The reliability of the NCMS was evaluated in terms of internal consistency and test–retest reliability following the establishment of structural validity. Internal consistency was assessed using multiple complementary indices, including Cronbach’s alpha coefficient, Spearman–Brown coefficient, Guttman split‐half reliability, item–total correlations and item discrimination analyses. Cronbach’s alpha values of ≥ 0.70 were considered acceptable, while values ≥ 0.80 indicated good internal consistency. Item–total correlation coefficients were examined to evaluate the contribution of each item to the overall scale, with values ≥ 0.30 considered acceptable. Items that did not meet this criterion were reviewed for potential revision or deletion. To further examine the stability of the scale over time, test–retest reliability was assessed. The NCMS was administered to a subgroup of 30 nurses at two different time points with a 2‐week interval, which is considered an appropriate time frame under COSMIN guidelines to minimise memory recall effects while assuming clinical stability of the target construct. Test–retest reliability was assessed using the intraclass correlation coefficient (ICC) based on a two‐way mixed‐effects model with absolute agreement for single measurements [ICC(3, 1)]. According to Koo and Li [36], ICC values between 0.75 and 0.90 indicate good reliability, whereas values greater than 0.90 indicate excellent reliability. Additionally, to ensure a multiperspective and comprehensive evaluation of temporal stability, a paired‐samples *t*‐test was conducted to confirm the absence of a significant mean difference between the two measurement points, and Pearson’s correlation coefficient (*r*) was calculated to verify strong linear association (*r* ≥ 0.85). In addition, measurement error was indirectly considered through the evaluation of test–retest reliability; however, standard error of measurement (SEM) and smallest detectable change (SDC) were not calculated, as the primary aim of this study was initial scale development rather than responsiveness analysis.

### 3.5. Participants and Sampling

Each phase of the study was conducted with different sample groups. The study included a total of 807 nurses working in a university hospital located in the city center of Trabzon. Among them, 30 nurses participated in the pilot study, 397 nurses were included in the PCA, and 350 nurses participated in the CFA. The same sample used in the CFA was also used for reliability analyses, while 30 nurses participated in the test–retest reliability assessment. In scale development studies, a sample size of at least 200–300 participants is generally considered adequate [20]. According to Comrey and Lee [37], sample sizes can be classified as 50 (very poor), 100 (poor), 200 (fair), 300 (good), 500 (very good) and 1000 (excellent). In addition, it has been suggested that a minimum of 30 participants is sufficient for pilot testing and test–retest procedures [38]. Based on these recommendations, the sample size used in this study was considered adequate for developing a scale with robust psychometric properties. Convenience sampling, a type of non‐probability sampling method, was used to recruit participants for the study (Table 2).

**TABLE 2:** Sampling strategy, sample sizes and characteristics across psychometric phases (*N* = 807).

Psychometric phase	Purpose	Participants	Sampling method	Sample size (n)	Justification
Phase 1	Pilot testing	Registered nurses	Convenience Sampling	30	≥ 30 participants recommended for pilot testing
Phase 2	Construct validity (PCA)	Registered nurses	Convenience Sampling	397	Adequate for construct validity (≥ 200–300)
Phase 3	Construct validity (CFA)	Independent sample of registered nurses	Convenience Sampling	350	Independent confirmation of factor structure
Phase 4	Internal consistency reliability	Same CFA sample	Convenience Sampling	350	Reliability analyses
Phase 5	Test–retest reliability	Subsample of registered nurses	Convenience Sampling	30	≥ 30 participants recommended for stability testing

*Note:* Recommendations are based on Boateng et al. [20], Comrey and Lee [37] and Tavşancıl [38].

The inclusion criteria for the study were being employed as a nurse at the relevant hospital for at least 1 month and providing voluntary consent to participate in the research.

The inclusion criteria for the study were being employed as a nurse at the relevant hospital for at least 1 month and providing voluntary consent to participate in the research. The exclusion criteria were working as a nurse in the administrative or outpatient (polyclinic) unit of hospital.

### 3.6. Data Collection and Procedure

Data were collected using a personal information form and the draft NCMS. The personal information form consisted of four questions addressing nurses’ sociodemographic characteristics, including age, gender, years of professional experience and clinical working unit. The draft scale was developed to determine the motivational factors and dimensions influencing nurses’ motivation to provide care. It was structured as a five‐point Likert‐type scale ranging from 1 to 5 (1 = strongly disagree, 2 = disagree, 3 = neutral, 4 = agree and 5 = strongly agree). Data were collected between December 2024 and October 2025 through questionnaires (personal information form and scale) distributed in person by the researchers to nurses working at a university hospital. A total of 870 questionnaires were distributed, of which 830 were returned. After excluding 13 questionnaires due to incomplete responses, the final sample consisted of 807 completed questionnaires. Institutional permission was obtained from the relevant university hospital prior to the data collection process. On average, participants required approximately 15–30 min to complete the data collection instruments.

### 3.7. Data Analysis

Statistical analyses were performed using SPSS Version 26.0 (IBM Corp., Armonk, NY, USA) and AMOS version 21.0 software. Descriptive statistics, including frequencies, percentages, means and standard deviations, were used to summarise demographic characteristics. Content validity was evaluated via I‐CVI, S‐CVI/Ave and modified kappa coefficients. Dataset suitability for factor analysis was verified using the KMO index and Bartlett’s test of sphericity. Construct validity was examined through PCA with Promax rotation and verified via CFA using ML estimation. Convergent and discriminant validity criteria were evaluated based on CR, AVE, MSV, ASV and HTMT ratios. Internal consistency was tested using Cronbach’s alpha, Spearman–Brown and Guttman split‐half coefficients. Temporal stability was analysed using ICC (3, 1), paired‐samples *t*‐tests and Pearson correlation coefficients. For all statistical tests, the level of significance was set at *p* < 0.05.

### 3.8. Ethical Considerations

Prior to the study, ethical approval was obtained from the Ethics Committee of a university (Approval No: E‐13562490‐150‐050.01.04‐527571; Date: 01.07.2024). Institutional permission was subsequently obtained from the relevant hospital (Approval No: E‐48814514‐299‐88747/E‐63582098‐299‐7906). Before data collection, nurses who met the inclusion criteria were informed about the purpose of the study, the data collection tools, and the estimated time required to complete the questionnaires. Participants were also assured that their personal information would remain confidential and that the collected data would be used solely for research purposes. Informed consent was obtained from all participants.

## 4. Results

Among the nurses included in the PCA, 86.6% were female. The mean age of participants was 32.43 ± 8.98 years, and the mean duration of professional experience was 10.25 ± 8.70 years. Regarding their clinical units, 53.7% worked in inpatient clinics, 37.8% in intensive care units, and 8.6% in outpatient clinics.

Among the nurses included in the CFA, 88.9% were female. Their mean age was 33.04 ± 9.17 years, and the mean duration of professional experience was 10.94 ± 9.00 years. In terms of clinical distribution, 55.4% worked in inpatient clinics, 35.7% in intensive care units and 8.9% in outpatient clinics. Before conducting PCA, the KMO measure was 0.948, and Bartlett’s test of sphericity was statistically significant (*χ* ^2^ = 8438.372, *p* < 0.001). The anti‐image correlation values ranged between 0.90 and 0.97, indicating that the data were appropriate for factor analysis. Subsequently, the scale underwent six rotations using PCA with the Promax rotation method. During PCA, the rotated pattern matrix was examined for cross‐loading items. Fourteen items were excluded because they either had factor loadings below 0.50 or exhibited substantial cross‐loadings across multiple factors, resulting in the final 27‐item, five‐factor structure. The final structure consisted of 27 items across five dimensions: SCC, PIV, PCO, SAC and SSS. These five factors had eigenvalues greater than 1 and explained between 12.4% and 16.2% of the variance individually. Together, they accounted for 72.35% of the total variance of the scale (Table 3). The scree plot also indicated a clear breakpoint after the fifth factor, supporting the five‐factor structure of the scale. The mean total score of the NCMS was 4.41 ± 0.51. The mean scores of the subdimensions were as follows: SCC 4.28 ± 0.73, PIV 4.51 ± 0.55, PCO 4.57 ± 0.51, SAC 4.16 ± 0.75 and SSS 4.50 ± 0.54.

**TABLE 3:** Subdimensions of the NCMS and factor loadings.

Item No	Subdimensions	Eigenvalue	Variance %	Mean	SD	Factor loadings
	*Factor 1: Supportive Care Conditions*	4.37	16.20	4.28	0.73	0.628–0.977
26	Availability of opportunities to provide care in an environment consistent with professional standards			4.24	0.90	0.898
27	The patient being ready to receive care			4.29	0.77	0.719
29	Availability of sufficient resources to provide care			4.29	0.87	0.977
30	Participation in decision‐making processes related to care			4.29	0.81	0.810
32	Recognition of the value of nursing care			4.36	0.89	0.849
34	Use of spiritual encouragement supporting the care provided			4.24	0.94	0.628

	*Factor 2: Professional Identity and Values*	4.18	15.51	4.51	0.55	0.641–0.949
36	Providing care consistent with professional values and beliefs			4.50	0.69	0.641
37	Gaining professional development and experience through caring			4.53	0.60	0.810
38	Providing care with a team that shares professional values and beliefs			4.50	0.64	0.949
39	Providing care with a collaborative team			4.55	0.64	0.911
40	Having autonomy/independent decision‐making while providing care			4.47	0.66	0.802
41	Positive influence of the provided care on professional image			4.53	0.65	0.776

	*Factor 3: Positive Care Outcomes*	3.98	14.76	4.57	0.51	0.759–0.947
2	The care I provide relieves the patient			4.61	0.57	0.887
3	Visible improvement in the patient as a result of the care I provide			4.58	0.59	0.947
7	Meeting the patient’s hygiene needs through the care I provide			4.53	0.63	0.793
8	Relieving the patient’s pain through the care I provide.			4.57	0.59	0.776
9	Receiving positive feedback from the patient regarding the care I provide			4.57	0.61	0.759

	*Factor 4: Sense of Achievement and Competence*	3.63	13.46	4.16	0.75	0.664–0.936
10	Desire/motivation to be the best in providing care			4.01	1.03	0.903
11	Being competent in providing care			4.35	0.76	0.696
12	Being sought after or consulted regarding care			4.21	0.89	0.936
13	Receiving appreciation for the care I provide			4.24	0.90	0.746
22	Achieving what my colleagues cannot in providing care			4.02	1.06	0.664

	*Factor 5: Sense of Spiritual Satisfaction*	3.35	12.41	4.50	0.54	0.712–0.873
16	The care I provide strengthens my spiritual dimension			4.53	0.63	0.851
18	Solving the patient’s problem through the care I provide			4.50	0.66	0.804
19	Seeing the patient’s satisfaction with the care received			4.52	0.62	0.873
20	Reducing the patient’s anxiety through the care I provide			4.46	0.66	0.766
24	Feeling peaceful or satisfied with myself after providing care			4.51	0.63	0.712

A moderate to high level of correlation was found between the total NCMS score and the subdimension scores (Table 4).

**TABLE 4:** Correlation values between the NCMS total and subscales scores for the PCA sample.

Subscales	SCC *r*	PIV *r*	PCO *r*	SAC *r*	SSS *r*
SCC	—	—	—	—	—
PIV	0.734^∗∗^	—	—	—	—
PCO	0.570^∗∗^	0.619^∗∗^	—	—	—
SAC	0.511^∗∗^	0.525^∗∗^	0.460^∗∗^	—	—
SSS	0.617^∗∗^	0.643^∗∗^	0.636^∗∗^	0.638^∗∗^	—
NCMS	0.862^∗∗^	0.859^∗∗^	0.767^∗∗^	0.773^∗∗^	0.840^∗∗^

Abbreviations: NCMS = Nursing Care Motivation Scale, PCO = Positive Care Outcomes, PIV = Professional Identity and Values, SAC = Sense of Achievement and Competence, SCC = Supportive Care Conditions, SSS = Sense of Spiritual Satisfaction.

^∗∗^ *p* < 0.01.

**FIGURE 1:**
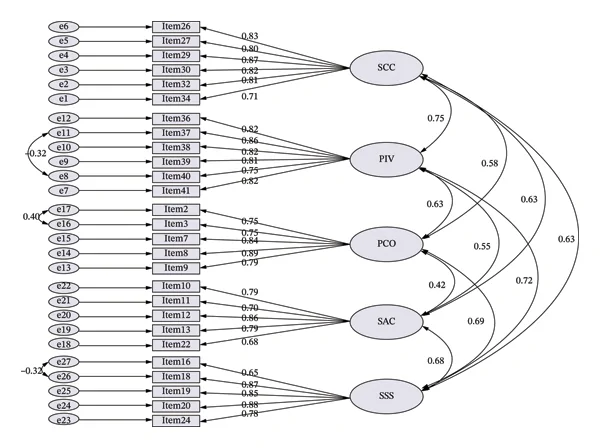
Path diagram of NCMS according to CFA results (F1: Supportive Care Conditions; F2: Professional Identity and Values; F3: Positive Care Outcomes; F4: Sense of Achievement and Competence; F5: Sense of Spiritual Satisfaction).

For construct validity, the KMO value of the scale in the pre‐CFA sample was 0.92, the Bartlett test was *χ* ^2^ = 5376.040; *p* < 0.001, and antiimage correlation values were found to be between 0.87 and 0.94. Subsequently, the five‐dimensional, 27‐item structure of the NCMS shaped by PCA was tested with CFA. Inspection of the modification indices indicated that model fit could be improved through three correlated error terms, all specified within, rather than across, subdimensions: between the error terms of Items 8 and 11 (e8–e11) within the PIV subdimension, Items 16 and 17 (e16–e17) within the PCO subdimension, and Items 26 and 27 (e26–e27) within the SSS subdimension. These three modifications were considered theoretically justified, as each pair of items belongs to the same latent factor and reflects closely related item content—for instance, Items 8 and 11 both address recognition‐related aspects of professional identity, Items 16 and 17 both reflect immediate patient‐level relief outcomes, and Items 26 and 27 both pertain to spiritual/emotional resolution following care—which is a commonly accepted rationale for allowing correlated measurement errors in CFA [39, 40]. No cross‐loadings or structural paths were modified. In the model with these three modifications, factor loading values were between 0.65 and 0.89 (Figure 1), and fit indices were found to be at an acceptable to good level (Table 5). The mean total NCMS score was 4.44 ± 0.44, and the subdimensions were 4.30 ± 0.64 for SCC, 4.56 ± 0.47 for PIV, 4.64 ± 0.41 for PCO, 4.21 ± 0.65 for SAC and 4.52 ± 0.52 for SSS.

**TABLE 5:** CFA fit index values of NCMS.

Index	Scale values	Good fit	Acceptable fit	Result
*χ* ^2^/sd	2.446	≤ 3	4–5	Good Fit
GFI	0.87	≥ 0.90	(0.89‐0.85)	Acceptable Fit
CFI	0.94	≥ 0.95	> 0.90	Acceptable Fit
RMSEA	0.06	≤ 0.05	(0.06‐0.08)	Acceptable Fit
RMR	0.02	< 0.05	< 0.08	Good Fit
IFI	0.94	> 0.95	> 0.90	Acceptable Fit
TLI	0.93	≥ 0.95	(0.94‐0.90)	Acceptable Fit
NFI	0.90	> 0.95	> 0.90	Acceptable Fit
SRMR	0.04	≤ 0.05	(0.06‐0.08)	Good Fit

*Note:* *X* ^2^/Sd: adjusted chi‐square statistic, GFI: fit index, NFI: normalized fit index [41].

Abbreviations: CFI = comparative fit index, IFI = incremental fit index, RMR = root mean square, RMSEA = root mean square errors of approximation, SRMR = standardized root mean square residual, TLI = Tucker–Lewis Index.

The CFA demonstrated an overall acceptable model fit. The *χ* ^2^/df, RMR and SRMR values indicated good model fit, while the GFI, CFI, RMSEA, IFI, TLI and NFI values indicated acceptable model fit. Overall, these findings support the adequacy of the proposed five‐factor measurement model and provide evidence for the construct validity of the NCMS [42, 43].

The AVE values ranged from 0.588 to 0.665, the CR values ranged from 0.876 to 0.922, the MSV values ranged from 0.456 to 0.560 and the ASV values ranged from 0.303 to 0.462 (Table 6). All constructs demonstrated adequate convergent validity, with AVE values exceeding 0.50 and CR values exceeding 0.70. Furthermore, AVE values were greater than both MSV and ASV for all constructs, and all HTMT values were below the recommended threshold of 0.85, providing evidence of adequate discriminant validity (Table 6).

**TABLE 6:** CR, AVE, MSV and ASV values of NCMS.

Subdimensions	CR	AVE	MSV	ASV	√AVE	HTMT				
						SCC	PIV	PCO	SAC	SSS
SCC	0.919	0.655	0.560	0.391	0.809	—				
PIV	0.922	0.664	0.560	0.446	0.815	0.766	—			
PCO	0.902	0.649	0.472	0.345	0.806	0.559	0.623	—		
SAC	0.876	0.588	0.456	0.303	0.767	0.568	0.574	0.441	—	
SSS	0.904	0.656	0.523	0.462	0.810	0.649	0.744	0.678	0.715	—

*Note:* HTMT = Heterotrait–Monotrait ratio of correlation.

Abbreviations: ASV = Average Shared Variance, AVE = Average Variance Extracted, √AVE = square root of the average variance extracted, CR = Composite Reliability, MSV = Maximum Shared Variance, NCMS = Nursing Care Motivation Scale, PCO = Positive Care Outcomes, PIV = Professional Identity and Values, SAC = Sense of Achievement and Competence, SCC = Supportive Care Conditions, SSS = Sense of Spiritual Satisfaction.

In the reliability analysis, the overall Cronbach’s alpha coefficient for the NCMS in the PCA sample was 0.955, with subdimension values ranging from 0.862 to 0.936. Item–total correlation coefficients ranged from 0.528 to 0.758 for the total scale. At the subdimension level, correlations ranged from 0.677 to 0.827 for SCC, 0.748 to 0.820 for PIV, 0.731 to 0.810 for PCO, 0.604 to 0.802 for SAC and 0.603 to 0.836 for SSS (*p* < 0.001). The Spearman–Brown coefficient for the total scale was 0.849, and the Guttman split‐half coefficient was 0.846. In the CFA sample, the overall Cronbach’s alpha coefficient was 0.952, with subdimension values ranging between 0.865 and 0.914. Item–total correlation coefficients ranged from 0.538 to 0.742 for the total scale. At the subdimension level, correlations ranged from 0.677 to 0.829 for SCC, 0.696 to 0.796 for PIV, 0.741 to 0.807 for PCO, 0.623 to 0.789 for SAC and 0.596 to 0.816 for SSS (*p* < 0.001). The Spearman–Brown coefficient for the total scale was 0.845, and the Guttman split‐half coefficient was 0.843. Comparison of the upper and lower 27% groups demonstrated statistically significant differences in the total NCMS score (*t* = −44.064, *p* < 0.001) as well as across all individual items (*p* < 0.001).

In addition, to determine the temporal stability of the NCMS, the 27‐item scale was administered to 30 nurses at 2‐3 week intervals. In the test–retest analysis, a high level of positive correlation was found between the total NCMS score and its subdimensions and the nurses’ initial and final test scores (*r* = 0.876‐0.955; *p* < 0.001), while no statistically significant difference was found between the mean scores (*t* = −1.448, *p* > 0.05). Furthermore, the ICC of the scale was 0.87 and above (Table 7).

**TABLE 7:** Intraclass correlation coefficient between NCMS test–retest scores.

Subdimension	*r*	Pretest mean ± SD	Posttest mean ± SD	*t*	*p*	ICC
SCC	0.923	4.13 ± 0.69	4.17 ± 0.70	−0.665	0.512	0.924
PIV	0.949	4.53 ± 0.48	4.57 ± 0.48	−1.366	0.182	0.947
PCO	0.876	4.66 ± 0.35	4.70 ± 0.34	−1.044	0.305	0.875
SAC	0.955	4.38 ± 0.56	4.42 ± 0.55	−1.293	0.206	0.954
SSS	0.926	4.47 ± 0.59	4.52 ± 0.54	−1.313	0.199	0.921
NCMS total	0.937	4.43 ± 0.41	4.47 ± 0.42	−1.448	0.158	0.934

*Note:* M, mean; *r*: Pearson’s correlation; *t*: paired‐sample t‐test.

Abbreviations: ICC = intraclass correlation coefficient, NCMS = Nursing Care Motivation Scale, PCO = Positive Care Outcomes, PIV = Professional Identity and Values, SAC = Sense of Achievement and Competence, SCC = Supportive Care Conditions, SD = standard deviation, SSS = Sense of Spiritual Satisfaction.

Based on the results of the validity and reliability analyses, the NCMS comprises 27 items organised into five subdimensions: supportive conditions in care, professional identity and values, PCO, SAC and sense of spiritual satisfaction. All items are positively worded, and the scale does not include any reverse‐coded items. The NCMS is a five‐point Likert‐type instrument, with response options ranging from 1 (strongly disagree) to 5 (strongly agree). Scale scores are calculated using the arithmetic mean. Interpretation of both total and subdimension scores is as follows: scores between 1.00 and 2.33 indicate low levels of caring motivation, scores between 2.34 and 3.66 indicate moderate levels, and scores between 3.67 and 5.00 indicate high levels of nursing care motivation.

## 5. Discussion

In the tool development stage, nursing care motivation was conceptually divided into five factors of “Supportive Care Conditions,” “Professional Identity and Values,” “Positive Care Outcomes for the Patient,” “Sense of Achievement and Competence” and “Sense of Spiritual Satisfaction”. In the factor analysis performed to test the structural validity, the items explicitly clustered into the intended five‐factor structure. These findings indicate that the proposed conceptual structure was empirically supported and that the theoretical domains underlying nursing care motivation were successfully operationalised into measurable constructs. Factor loadings of 0.50 or higher indicate that the items adequately represent the underlying construct measured by the scale [20, 44]. In addition, the total explained variance exceeding 70%, with each of the five subdimensions accounting for more than 12% of the variance, provides strong support for the proposed five‐factor structure and indicates that the model explains the construct comprehensively. This substantial explanatory power demonstrates that the conceptually generated factors successfully retained their structural integrity during the empirical validation stage. Importantly, the empirical factor structure closely reflected the theoretical framework established during instrument development, supporting both the conceptual coherence and construct validity of the NCMS. During the exploratory factor analysis, several items were removed because they did not satisfy the predefined psychometric criteria, including factor loadings below 0.50 [28, 45] or substantial cross‐loadings across multiple factors. These findings suggested that the removed items either failed to uniquely represent a single latent construct or provided conceptually redundant information. Although the number of items was reduced, item removal was guided by both psychometric and theoretical considerations, ensuring that each retained factor continued to adequately represent its intended conceptual domain. Consequently, the refinement process improved the psychometric performance of the NCMS while preserving its theoretical integrity. Thus, the item refinement process strengthened the psychometric performance of the scale while preserving its conceptual foundation. This finding exceeds commonly recommended thresholds, which suggest that total explained variance in multidimensional scales should range between 40% and 60%, with at least 5% explained variance per factor considered acceptable [30, 41]. Moreover, the Cronbach’s alpha coefficients obtained for the five‐factor structure in the PCA sample were all above 0.70, indicating a high level of internal consistency for the NCMS [46]. Following the PCA, the five‐factor structure of the NCMS was further evaluated using CFA [47]. The CFA results indicated that commonly reported model fit indices (*χ* ^2^/SD, GFI, CFI, RMSEA, SRMR, NFI and TLI) were within acceptable or good ranges [33], thereby supporting the adequacy of the proposed five‐dimensional model. Although the GFI value (0.87) was slightly below the conventional threshold of 0.90, values of ≥ 0.85 have also been considered indicative of acceptable model fit [42, 43]. The relatively lower GFI value may be attributed to the multidimensional structure of the NCMS and the sensitivity of the GFI index to model complexity. In the present study, the GFI therefore indicated acceptable model fit. Moreover, model fit should be interpreted using multiple complementary fit indices rather than relying on a single index. The remaining fit indices consistently demonstrated acceptable to good model fit, further supporting the adequacy of the proposed measurement model. Furthermore, theoretically justified model modifications, guided by the modification indices, were implemented during the CFA to improve model fit. These theoretically justified modifications improved model fit without altering the conceptual structure of the NCMS, further supporting the consistency between the proposed theoretical framework and the final measurement model. Furthermore, the findings satisfied established criteria for convergent and discriminant validity. All AVE values exceeded 0.50, CR values exceeded 0.80, and the criterion of CR > AVE was met across all dimensions. In addition, the AVE of each factor was greater than the corresponding MSV and ASV values, while all HTMT ratios remained below the recommended threshold, confirming that the five subdimensions represent related yet empirically distinct aspects of nursing care motivation [45]. These findings support the construct validity of the NCMS and indicate that separate scores can be meaningfully calculated and interpreted for each subdimension. Although the present findings provide strong evidence supporting the proposed five‐factor structure, nursing care motivation is inherently influenced by organisational, cultural and clinical contexts. Therefore, future studies should examine the stability of the NCMS across different healthcare settings, nursing specialties and cultural populations using measurement invariance and cross‐validation analyses. Such evidence would further strengthen the generalisability and robustness of the proposed measurement model.

Following CFA, the high Cronbach’s *α* coefficient (> 0.85), along with Spearman–Brown and Guttman split‐half coefficients exceeding 0.80, indicates that the scale demonstrates strong internal reliability [46]. Item–total correlation coefficients above 0.50 further support the internal consistency of the scale and confirm that each item contributes meaningfully to the overall construct [45]. Additionally, the statistically significant differences observed between the upper and lower 27% groups in both total and subdimension scores indicate that the NCMS has strong discriminative power, effectively distinguishing between individuals with low and high levels of nursing care motivation [47]. This capacity proves that the individual items are highly sensitive to variations in nurses’ inner professional drives. Furthermore, test–retest analyses indicate that the NCMS provides stable and consistent measurements over time, yielding similar responses across administrations and demonstrating resistance to random measurement error [38]. This is evidenced by a strong, positive, and statistically significant correlation (*r* > 0.85) between two measurements conducted 2 weeks apart, alongside the absence of any significant difference between the mean scores of the two administrations, confirming the exceptional temporal stability of the tool in dynamic clinical environments. Unlike existing instruments that focus narrowly on general work motivation or isolated caring behaviours, the NCMS conceptualises nursing care motivation as a multidimensional construct integrating organisational, professional, patient‐related, competence‐based and spiritual dimensions. By operationalising these complementary domains within a single measurement model, the NCMS captures the complex synergistic mechanisms that drive nurses’ willingness to deliver high‐quality patient care. The five‐dimensional structure of the NCMS is consistent with contemporary nursing literature, which conceptualizes nursing care motivation as a multidimensional construct influenced by both organisational and personal factors. Previous studies have shown that supportive work environments and professional autonomy enhance nurses’ work engagement and quality of care [48–50], whereas professional identity strengthens caring behaviours and professional commitment [51]. Likewise, self‐efficacy and professional competence are associated with greater motivation [52], improved clinical performance and safer patient care [53]. Furthermore, positive patient outcomes and deriving emotional meaning from caring contribute to compassion satisfaction [54], resilience [55] and sustained professional motivation [56]. Accordingly, the five dimensions of the NCMS encompass the principal determinants of nursing care motivation identified in the literature, providing a theoretically grounded and comprehensive framework for assessing nurses’ motivation to deliver high‐quality care.

From a deeper analytical perspective, the five dimensions of the NCMS do not merely represent isolated psychometric factors but rather an interconnected system of motivational drivers within clinical environments. Specifically, the “Supportive Care Conditions” dimension reflects the structural foundation of practice, suggesting that administrative backing directly dictating nurses’ functional capacity. Concurrently, the “Professional Identity and Values” and “Sense of Achievement and Competence” dimensions represent internal drivers, demonstrating that clinical expertise and internalised professional ethics foster a proactive standard of care. Finally, the “Positive Care Outcomes for the Patient” and “Sense of Spiritual Satisfaction” dimensions capture the relational and existential rewards of nursing. This structural synergy implies that when healthcare organisations strengthen structural support, it directly activates nurses’ internal professional values, resulting in superior patient outcomes and sustainable care motivation.

Furthermore, the study revealed that nurses in both the PCA and CFA samples obtained high mean scores on the NCMS, indicating that their motivation and willingness to provide care were generally high, both overall and across subdimensions. Notably, subdimension scores did not follow a uniform pattern, with “Positive Care Outcomes for the Patient” yielding the highest mean score (*M* = 4.57, SD = 0.51) and “Sense of Achievement and Competence” the lowest (*M* = 4.16, SD = 0.75). This suggests that nurses’ motivation is more consistently sustained by the relational and outcome‐oriented rewards of caregiving than by a stable sense of professional achievement. Within the Turkish healthcare context, characterised by nurse‐to‐population ratios substantially below the OECD average and persistently demanding workload conditions [57], such structural constraints may partly explain this comparatively lower and more variable score. These findings underscore the utility of interpreting NCMS subdimension profiles individually, rather than relying solely on the total score, to guide targeted institutional interventions.

These findings also provide preliminary evidence that the NCMS is capable of capturing motivational characteristics within clinical nursing populations, supporting its potential applicability in both research and practice. This finding aligns with prior research using similar instruments, which has consistently reported moderate to high levels of care‐oriented behaviours among nurses. Research employing the Caring Behaviours Inventory to evaluate caring behaviours has consistently reported that nurses score at moderate‐to‐high levels, indicating generally positive attitudes toward the delivery of care [58]. The conceptual domains represented in established caring instruments substantially overlap with several dimensions of the NCMS. For instance, technical competence, professional presence and emotional support—frequently reported as essential components of caring behaviours—correspond closely to the “Sense of Achievement and Competence” and “Professional Identity and Values” dimensions of the present scale.

The literature highlights that job motivation is a critical determinant of nurses’ care behaviours [59], with highly motivated nurses demonstrating greater engagement in care processes [60] and more effectively implementing compassionate and holistic care practices [3]. Similarly, Veenstra et al. [61] reported a positive association between work motivation and care outcomes, indicating that higher motivation levels correspond to improved care quality and nursing performance. These findings may be explained by the multidimensional nature of nursing care motivation captured by the NCMS. Nurses who perceive supportive care environments, internalise professional values, experience competence in clinical practice, observe positive patient outcomes and derive spiritual fulfillment from caring are more likely to sustain high levels of motivation. This interpretation is consistent with SDT, which emphasises the importance of autonomy, competence and relatedness in fostering intrinsic motivation [62], as well as Watson’s Theory of Human Caring, which highlights the moral, relational and transpersonal dimensions of nursing care. Collectively, these findings support the theoretical relevance of the NCMS dimensions and reinforce the relationship between nursing care motivation and high‐quality caring practices. Crucially, the inclusion and empirical validation of the “Sense of Spiritual Satisfaction” dimension extends the theoretical depth of the NCMS beyond traditional occupational motivation frameworks. In high‐stress clinical environments, care motivation often wanes due to systemic overload and compassion fatigue. By capturing the existential meaning and spiritual fulfillment derived from therapeutic human‐to‐human connections—as posited by Watson’s Caring Theory—this dimension explains why some nurses maintain high care motivation despite adverse workplace conditions. This deeper insight highlights that spiritual fulfillment acts as a vital psychological buffer against burnout, transforming routine care delivery into a deeply meaningful professional commitment. Taken together, these findings suggest that nursing care motivation is not merely an occupational characteristic but a multidimensional construct encompassing organisational, professional, clinical, relational and humanistic components.

Beyond its strong psychometric properties, the NCMS has important implications for nursing management, education and research. Its multidimensional structure enables nurse managers and educators to identify specific domains contributing to low nursing care motivation rather than relying solely on an overall motivation score. For example, lower scores in the “Supportive Care Conditions” dimension may indicate organisational barriers requiring improvements in leadership, staffing or workplace support, whereas lower scores in the “Professional Identity and Values” or “Sense of Achievement and Competence” dimensions may highlight the need for professional development, mentorship, or competency‐based educational interventions. Similarly, the “Positive Care Outcomes for the Patient” and “Sense of Spiritual Satisfaction” dimensions provide insight into nurses’ intrinsic motivational resources, which are closely associated with compassionate care, professional fulfillment, resilience and long‐term retention. Consequently, the NCMS serves not only as a robust outcome measure for intervention studies but also as a practical diagnostic tool for monitoring care motivation and evaluating organisational initiatives. By moving beyond global motivation scores, the scale enables healthcare managers to pinpoint specific domain‐level deficits and execute evidence‐based, targeted strategies that simultaneously strengthen nursing staff well‐being and clinical care quality. In terms of policy and executive leadership, these findings offer actionable strategies for nurse executives aiming to mitigate turnover and optimise retention. Rather than applying broad, one‐size‐fits‐all incentive programmes, hospital administrators can utilise domain‐specific NCMS profiles to design precision management strategies. For instance, if an audit reveals diminished scores in “Sense of Achievement and Competence,” nursing managers can implement structured clinical mentorship, clinical simulation training and autonomy‐enhancing protocols. Conversely, if “Supportive Care Conditions” yields low scores, executive attention can be immediately directed toward restructuring nurse‐to‐patient ratios, optimising shift scheduling and fostering transparent leadership channels. Thus, the NCMS transitions from a theoretical measurement tool to an indispensable diagnostic instrument for organisational development and clinical governance.

The NCMS also addresses an important gap in the existing literature. Although several instruments assess general work motivation, work engagement, professional values or caring behaviours among nurses, none comprehensively evaluates the multidimensional motivational processes underlying nursing care delivery. By integrating organisational, professional, clinical, patient‐related and spiritual dimensions into a single theoretical framework, the NCMS provides a comprehensive and nursing‐specific assessment of care motivation that extends beyond existing measures of work motivation and caring behaviours. This conceptual specificity offers researchers and clinicians a theoretically grounded instrument for investigating the determinants and outcomes of nursing care motivation across diverse clinical settings. Furthermore, the scale may facilitate the evaluation of interventions aimed at enhancing nurses’ motivation, ultimately contributing to improved quality of care, professional well‐being and patient outcomes. Future studies should investigate whether NCMS scores predict clinically relevant outcomes such as nurse retention, burnout, patient safety indicators, quality of care and organisational commitment. Longitudinal studies may also determine whether changes in nursing care motivation precede improvements in patient outcomes over time.

### 5.1. Limitations

There are some limitations to this study that are worth discussing. Firstly, the use of convenience sampling may introduce selection bias, potentially limiting the representativeness of the broader nursing population. Secondly, the NCMS was validated in a single hospital, which may restrict its applicability to other healthcare environments and specialised clinical units. Thirdly, since the NCMS is self‐reported, social desirability bias is a potential limitation. To minimise this risk, complete anonymity was guaranteed, no identifying or institutional information was collected and participants were explicitly assured that their responses would remain strictly confidential and would never be used for performance evaluations. In addition, because the present study focused primarily on structural validity and reliability, future studies should further examine the predictive validity, responsiveness and measurement invariance of the NCMS across different healthcare systems and cultural contexts. Finally, although the scale is theoretically designed for diverse nursing contexts, further research is needed to implement and validate the NCMS across varied clinical and cultural settings to enhance external validity and generalisability. Furthermore, the multidimensional structure of the NCMS allows healthcare organisations to monitor changes in specific motivational domains over time and evaluate the effectiveness of organisational, educational and leadership interventions aimed at strengthening nursing care motivation.

### 5.2. Implications for Nursing Management

The NCMS offers a practical tool for the accurate, consistent and multidimensional assessment of nursing care motivation and the factors influencing it. Regular measurement and monitoring of nurses’ motivation levels can inform institutional policies and managerial strategies, enabling targeted improvements. Training programmes and scientific activities addressing nursing care motivation can be redesigned and their effectiveness systematically evaluated. By identifying strengths, gaps and areas for improvement, the NCMS can support initiatives aimed at enhancing professional commitment, job satisfaction and care quality. Additionally, the scale provides valuable data for researchers conducting analytical studies, systematic reviews or meta‐analyses and can serve as a methodological guide for future research.

## 6. Conclusion

The NCMS was developed to assess nurses’ motivation and willingness to provide care, as well as the underlying motivational factors and dimensions guiding this behaviour. The scale demonstrates a multidimensional structure, comprising five subdimensions and 27 items: supportive conditions in care, professional identity and values, PCO, sense of achievement and competence and SSS. CFA supported this structure, revealing acceptable model fit indices. Reliability analyses indicated high internal consistency and stable measurements over time. Overall, the NCMS is a valid and reliable tool capable of evaluating nurses’ motivation to provide care in a multidimensional manner. According to this scale, nurses in both study samples exhibited high levels of care motivation, suggesting that caring remains a core intrinsic driver for nurses practising in clinical settings. These findings suggest that the NCMS is capable of capturing meaningful variation in nursing care motivation while remaining sensitive to the multidimensional nature of caring. Accordingly, the scale may facilitate future research examining the relationships between nursing care motivation and important outcomes such as quality of care, patient safety, professional well‐being, job retention and organisational performance across different clinical settings.

## Author Contributions

Yeter Kurt: conceptualization, methodology, formal analysis, software, validation, investigation, data curation, writing–original draft, writing–review and editing, project administration and supervision. Ebru Turhal: conceptualization, methodology, formal analysis, software, validation, investigation, data curation, writing–original draft and writing–review and editing. Havva Öztürk: conceptualization, methodology, validation, investigation, data curation, writing–review and editing and supervision. Aleyna Bülbül: investigation, data curation, methodology, validation and writing–review and editing.

## Funding

This research did not receive any specific grant from funding agencies in the public, commercial or not‐for‐profit sectors.

## Ethics Statement

This study was approved by the Karadeniz Technical University Health Sciences Scientific Research Ethics Committee (Ethical Approval Number: E‐13562490‐150‐050.01.04‐527571). Informed consent was obtained from all participants prior to their involvement in this study.

## Conflicts of Interest

The authors declare no conflicts of interest.

## Supporting Information

Additional supporting information can be found online in the Supporting Information section.

## Supporting information


**Supporting Information** STROBE‐checklist.

## Data Availability

The data that support the findings of this study are available on request from the corresponding author. The data are not publicly available due to privacy or ethical restrictions.
